# A *Bacillus thuringiensis *isolation method utilizing a novel stain, low selection and high throughput produced atypical results

**DOI:** 10.1186/1471-2180-5-52

**Published:** 2005-09-24

**Authors:** Joanne Rampersad, David Ammons

**Affiliations:** 1 Department of Life Sciences, The University of the West Indies, St. Augustine, Trinidad, Trinidad and Tobago; 2 The School of Veterinary Medicine, The University of the West Indies, Mt. Hope, Trinidad, Trinidad and Tobago

## Abstract

**Background:**

*Bacillus thuringiensis *is a bacterium known for producing protein crystals with insecticidal properties. These toxins are widely sought after for controlling agricultural pests due to both their specificity and their applicability in transgenic plants. There is great interest in isolating strains with improved or novel toxin characteristics, however isolating *B. thuringiensis *from the environment is time consuming and yields relatively few isolates of interest. New approaches to *B. thuringiensis *isolation have been, and continue to be sought. In this report, candidate *B. thuringiensis *isolates were recovered from environmental samples using a combination of a novel stain, high throughput and reduced selection. Isolates were further characterized by SDS-PAGE, light microscopy, PCR, probe hybridization, and with selected isolates, DNA sequencing, bioassay or Electron Microscopy.

**Results:**

Based on SDS-PAGE patterns and the presence of *cry *genes or a crystal, 79 candidate, non-clonal isolates of *B. thuringiensis *were identified from 84 samples and over 10,000 colonies. Although only 16/79 (20%) of the isolates showed DNA homology by Probe Hybridization or PCR to common *cry *genes, initial characterization revealed a surprisingly rich library that included a putative nematocidal gene, a novel filamentous structure associated with a crystal, a spore with spikes originating from a very small parasporal body and isolates with unusually small crystals. When compared to reports of other screens, this screen was also atypical in that only 3/79 isolates (3.8%) produced a bipyramidal crystal and 24/79 (30%) of the isolates' spores possessed an attached, dark-staining body.

**Conclusion:**

Results suggest that the screening methodology adopted in this study might deliver a vastly richer and potentially more useful library of *B. thuringiensis *isolates as compared to that obtained with commonly reported methodologies, and that by extension, methodologies fundamentally different from current methods should also be explored.

## Background

*Bacillus thuringiensis *is a bacterium known for producing protein crystals with pesticidal properties. These toxins are widely sought after for controlling agricultural pests due to both their specificity and their applicability in transgenic plants. Although any particular toxin has a desirably restricted host range, there is a large number of different toxins, each showing toxicity to one of many diverse pests. For these reasons there is currently great interest in isolating novel strains of *B. thuringiensis *with either unique host specificity or elevated toxicity. There are numerous reports on attempts to isolate novel *B. thuringiensis *strains from the environment [1,2]. In many of these, some form of enrichment step is used in conjunction with Phase Contrast Microscopy as the backbone of the screening strategy [3,4]. The results of these screens have a curiously similar result where a large proportion of the isolates (33.5% to 98%) contain a *cry1 *gene or a bipyramidal shaped crystal (known for toxicity against lepidopteran pests), [1,5,6] and relatively few, if any, novel or uniquely useful isolates are found. Herein we report on a screen for environmental isolates of *B. thuringiensis *on the Caribbean island of Trinidad, where a novel stain was used as an alternative to Phase Contrast Microscopy in a high-throughput method coupled with reduced selection.

## Results

### Isolation of native strains

**Figure 1:**
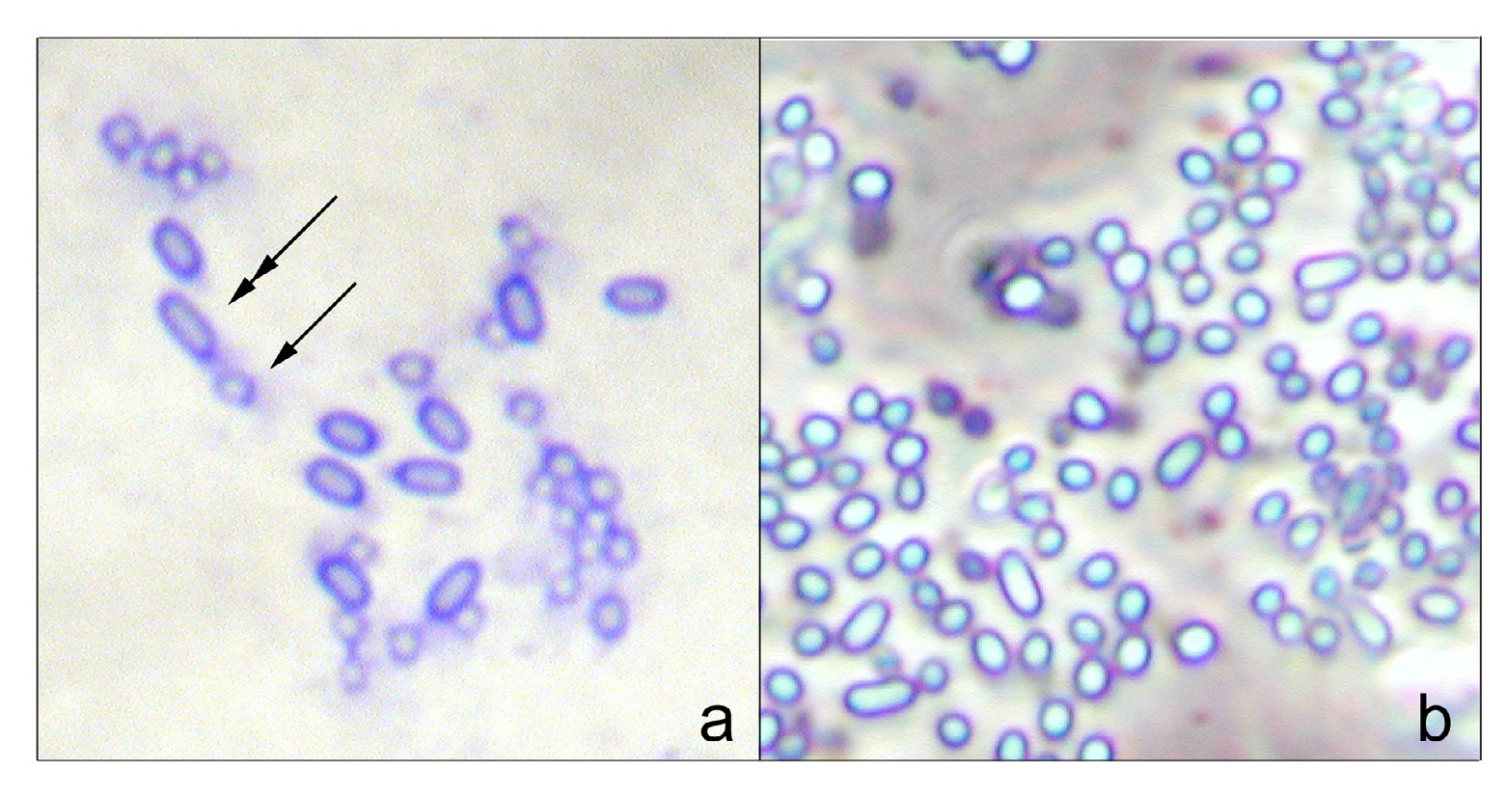
**Poorly staining crystals. **Stained (a) and Phase Contrast (b) micrographs of Bt1-32 showing spores (double arrow) and relatively lightly staining crystals (single arrow).

**Figure 2:**
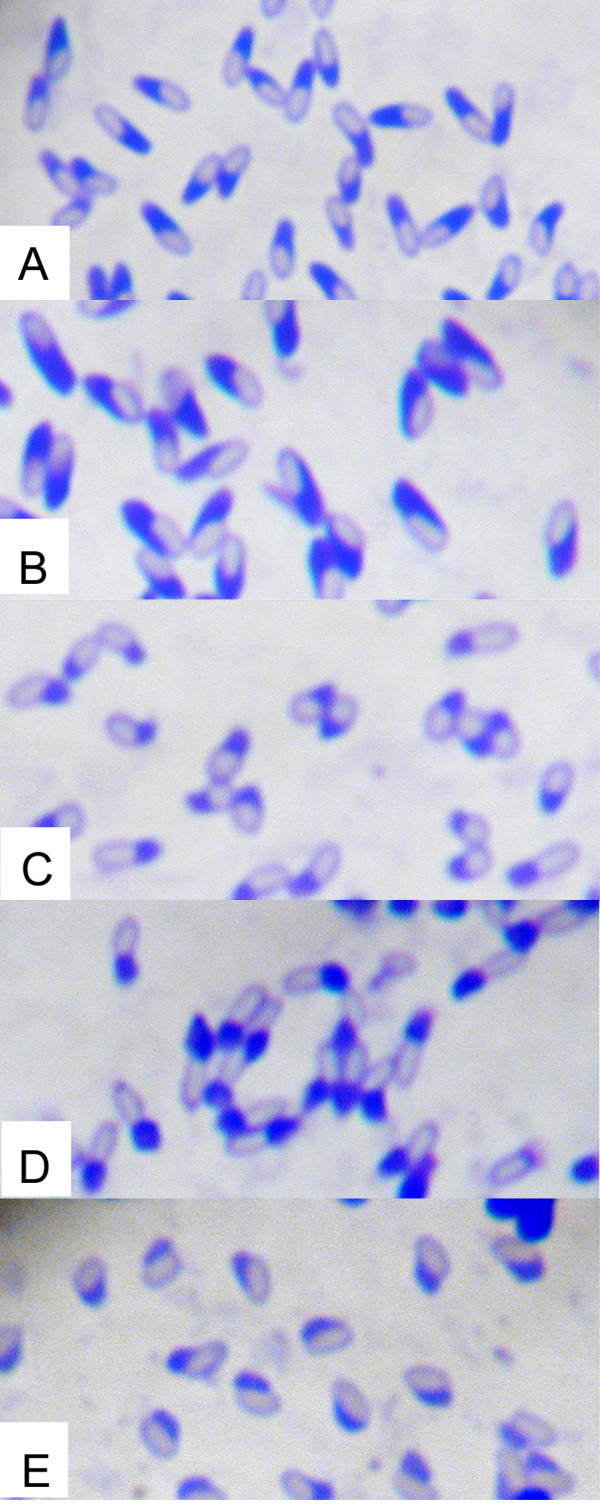
**Examples of the "capped" spore morphology. **Dark blue "caps" attached to lighter staining spores. A) Bt2-2, B) Bt1-48, C) Bt2-56, D) Bt1-46 and E) Bt2-14.

The use of a gridded-slide and a stain allowed a quick and high throughput assessment of 10,349 colonies from 84 samples. The preparation method was relatively quick, requiring approximately 15 min to grid a slide of 60 colonies and 3–5 s to evaluate each stained specimen on the grid. Evaluation could be done in a continuous motion without stopping to view each specimen. The stain also offered improved resolution over Phase Contrast, allowing the visualization of very small parasporal bodies [7]. A few of the crystals did not take up the stain well, however the contrast between spores and crystals was sufficient to easily differentiate the two, even when crystal morphology mimicked that of spores (Figure 1). The spores of 24/79 (30%) isolates were characterized as containing a dark-staining body which appeared as a "cap" on the spore (Figure 2) and which persisted after sporulation. Some of the caps may have contained crystals since oval or amorphous, phase dark objects with a light center, characteristic of crystals, were observed in 16/24 capped isolates by Phase Contrast Microscopy, and SDS-PAGE analysis showed protein bands for most of the capped isolates (data not shown).

Classifying isolates as clonal (siblings) if they were from the same sample and had the same crystal or cap morphology, 79 non-clonal isolates were collected comprising mostly round or amorphous crystals with 6 rectangular and 3 bipyramidal morphologies. Some samples were found to have multiple candidates of *B. thuringiensis*, either of the same or different parasporal body morphology.

**Figure 3:**
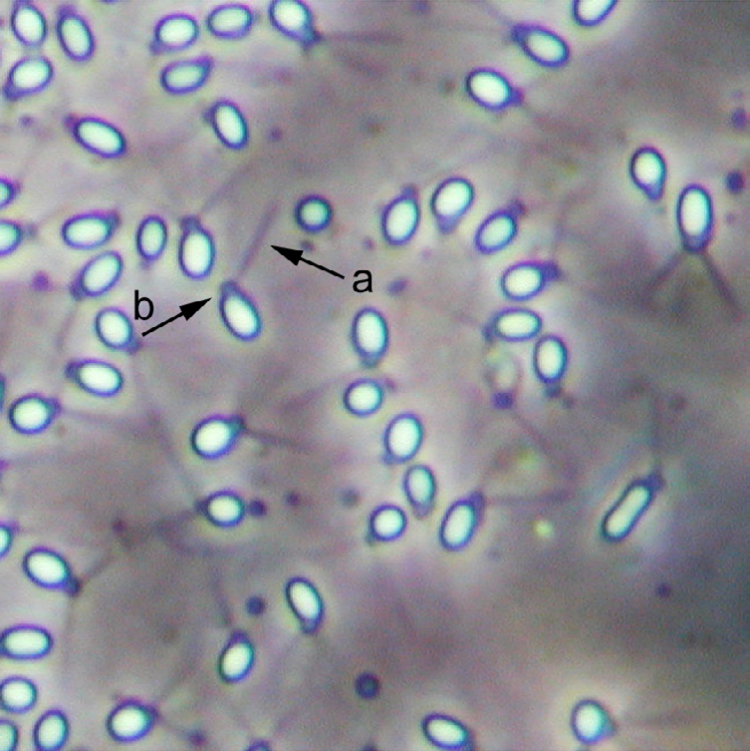
**Phase contrast micrograph showing spiked appendages emanating from the spores of Bt1-88. **Long straight spikes (a) emanating from a small, spore-associated parasporal body (b).

**Figure 4:**
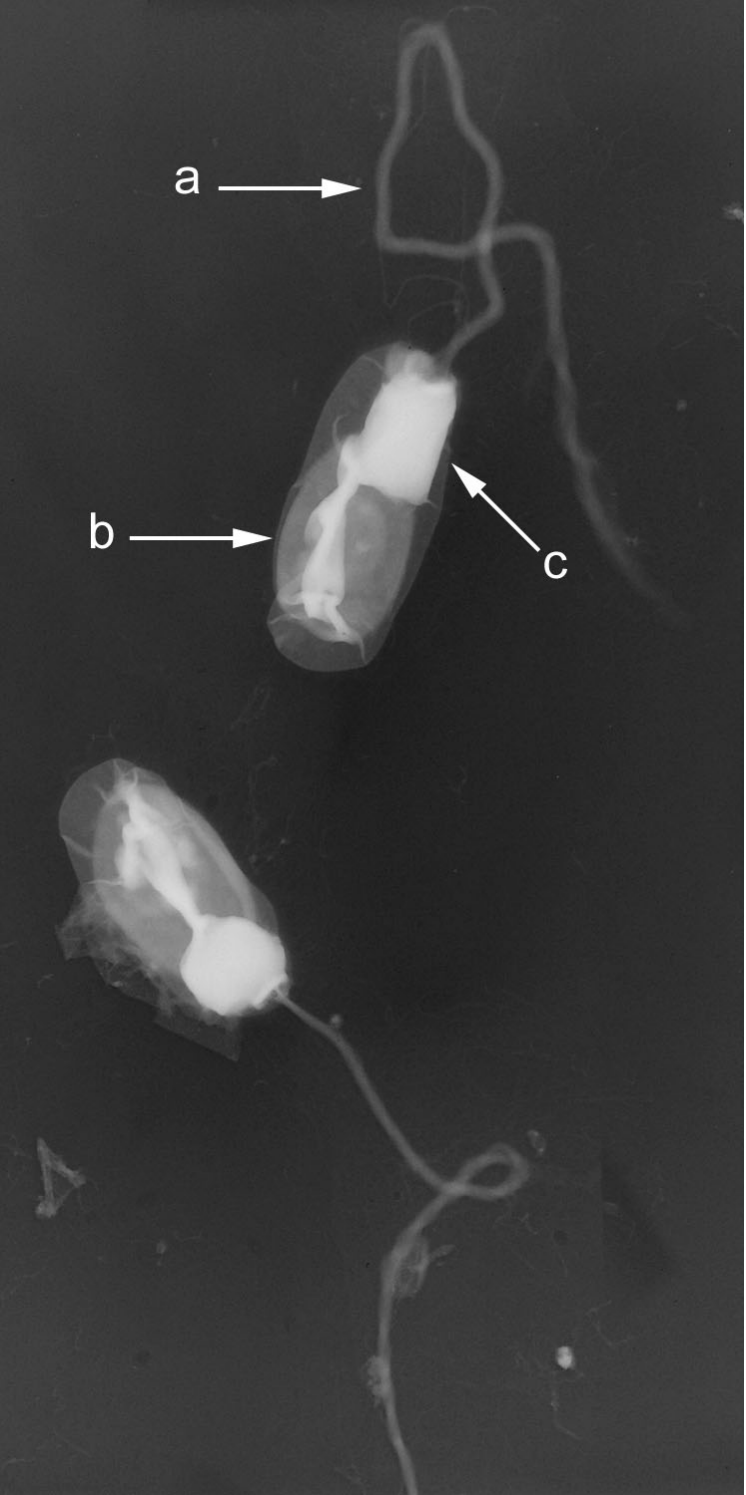
**Electron Micrograph of Bt2-56. **A Transmission Electron Micrograph of negatively stained spores from Bt2-56 containing a filament (a), and a sac-like structure containing a spore (b) and parasporal body (c).

Two isolates (Bt1-88 and Bt2-56), showed appendages emanating from parasporal bodies. Bt1-88 was characterized generally as having multiple, perhaps three, long spikes emanating from a small spore-associated parasporal body (Figure 3). SDS-PAGE analysis of Bt1-88 revealed two pronounced low molecular weight proteins of approximately 22 and 23 kDa (data not shown). Isolate Bt2-56 was found by electron microscopy to contain a multi-fiber filament emanating from a parasporal body located within a sack-like structure presumed to be the exosporium (Figure 4). A preliminary report on this isolate has been presented elsewhere, [8].

**Figure 5:**
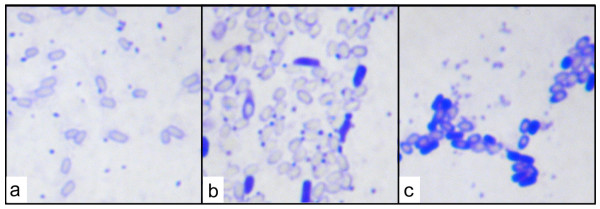
**Relatively small parasporal bodies. **Stained Brightfield micrographs (100X) of Bt1-22 (a), Bt1-40 (b) and Bt1-90 (c).

Three samples produced isolates with relatively small parasporal bodies, Bt1-26, Bt1-40 and Bt1-90 (Figure 5). Bt1-26 was isolated from sand taken a few inches above a high tide mark on a beach, Bt1-90 from a fresh manure pile (calf, sheep and goat), and Bt1-40 from composting manure collected from a barn housing sheep, goats, cattle, horses and pigs. SDS-PAGE analysis showed major protein bands of a relatively low molecular weight, between 11–19 kDa for each of these isolates (data not shown).

**Figure 6:**
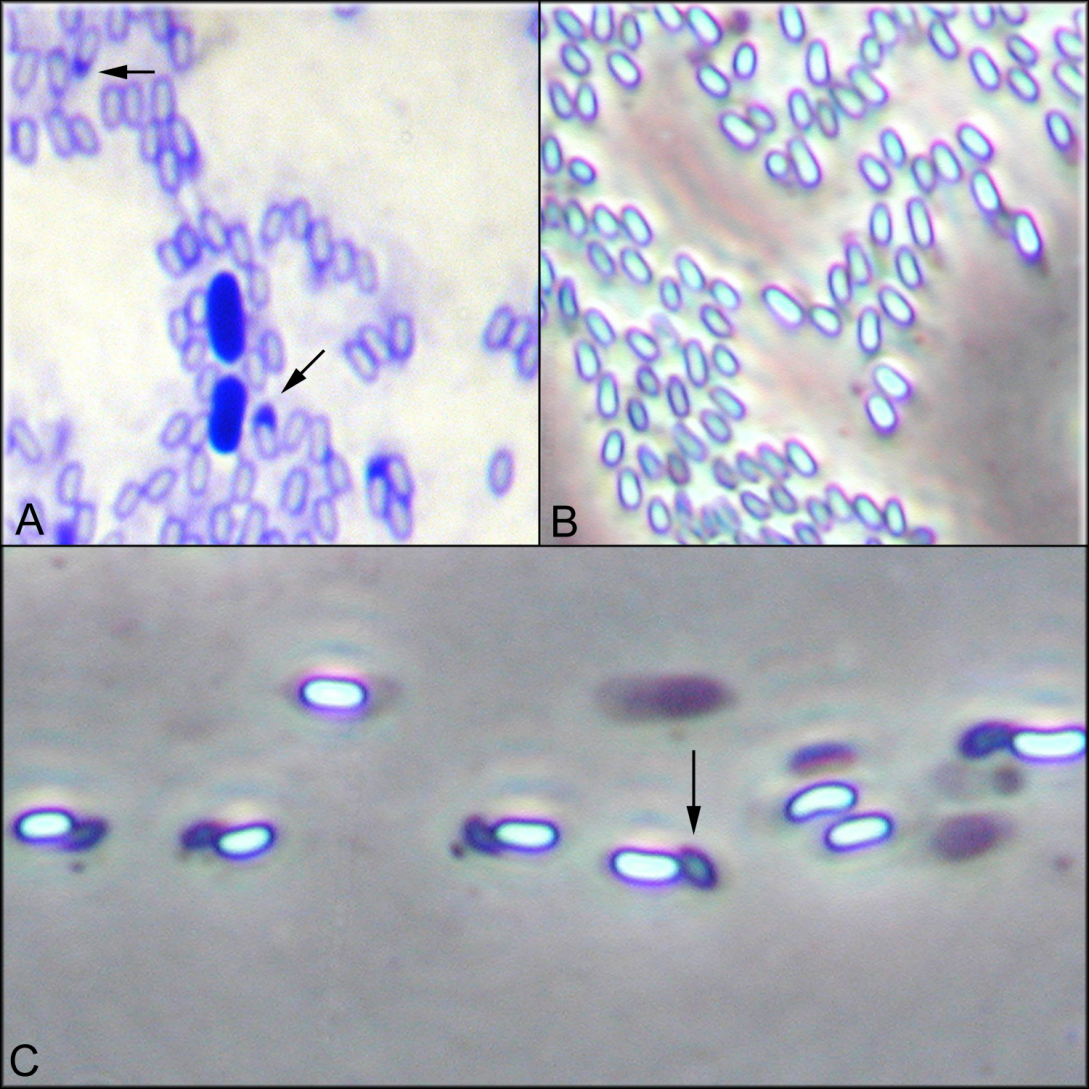
**Phase Contrast Microscopy of sporulated cells of Bt1-33. **A) stained preparation of Bt1-33 viewed under Brightfield microscopy, 100x oil immersion, B) Bt1-33 viewed under Phase Contrast microscopy, 100x oil immersion, C) Heat-treated preparation of Bt1-33 showing phase-dark crystals lying slightly lateral and terminal to the spore, Phase Contrast microscopy, 100x oil immersion.

Two isolates (Bt1-33 and Bt1-35) showed sequence homology to a nematocidal gene (See "*cry *gene profile" below). Bt1-33 showed no crystals and, among hundreds of spores within the microscopic field of vision, only a few possessed a blue-staining cap on one end of the spore. After repeated culture on media, the relative number of caps appeared to increase slightly (Figure 6A), which most probably would not have been detected by Phase Contrast Microscopy (Figure 6B). Crystals were not initially observed with either Phase Contrast Microscopy or stained specimens. However when specimens were heated strongly on the microscope slide, some specimens prepared in this manner showed oval crystals lying just lateral to the terminal end of the spore (Figure 6C). Interestingly, both Bt1-33 and Bt1-35 came from the same sample, an area of decomposing animal manure. Bt1-35 demonstrated a very small cap, however major protein bands were not observed on SDS gel analysis.

### *cry*-*cyt *gene profile

**Figure 7:**
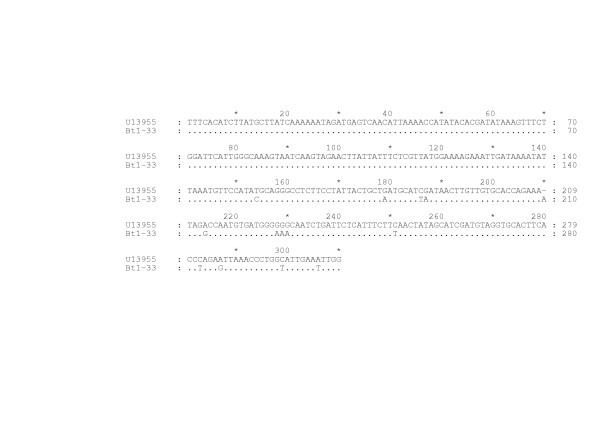
**DNA sequence of PCR amplicon from Bt1-33**. Comparison of the DNA sequence from Bt1-33 produced with the *cry5 *group primers and that of a known nematocidal gene, *cry14Aa*1 (GenBank accession number U13955). A "." is placed at those positions in Bt1-33 where the nucleotide is the same as that in U13955, and a letter denoting the nucleotide if it is not. Dashes "-" represent insertions required to align the sequences.

Probe and PCR analysis indicated that of the 79 non-clonal isolates, only 16 showed homology to any one of the major groups of *cry *genes tested (Table 1). *cry *and *cyt *genes were not detected in the capped isolates by either PCR or Probe Hybridization, except for Bt2-14, which was positive by PCR for a *cry4 *and *cyt2 *gene (Table 1). PCR amplification of Bt1-33 with primers showing homology to known nematocidal genes produced an amplicon of the expected size which, when sequenced, showed strong homology to a known nematocidal gene (GenBank accession number U13955, *cry14Aa*1) (Figure 7). However, Bt1-33 contains a mutation causing a frame shift, creating a stop codon. The amplified fragment from Bt1-33 was also used to probe the other *B. thuringiensis *candidates, only one of which, Bt1-35, also gave a weak but unquestionable hybridization signal (data not shown).

### Bioassay (quick screen)

A "quick screen" using relatively large amounts of sporulated colonies was used to ascertain if any of the isolates demonstrated appreciable activity against the 5^th ^instar larvae of agricultural pest, *Spodoptera frugiperda*. For most isolates, leaf pieces were completely eaten and given a score of 1.0 (non-toxic). Only one isolate and three control strains achieved a score greater than 2 and were considered toxic, (Bt2-46, with bipyramidal morphology and a score of 2.7; BGSC4A3, *B. thuringiensis *serovar *thuringiensis *HD2, Score 2.0; BGSC4D1, *B. thuringiensis *serovar *kurstaki *HD1, Score 2.0 and BGSC4J3, *B. thuringiensis *serovar *aizawai *HD133, Score 2.7).

## Discussion

With few exceptions [9,10] the crystal and spore of *B. thuringiensis *are released separately during sporulation. It was thus surprising that 24/79 non-clonal, candidate *B. thuringiensis *isolates possessed spores with a "capped" appearance. Some of these caps appeared to contain crystals since phase dark objects with a light center could be seen in many of the caps, including Bt2-56, which was subsequently confirmed by Electron Microscopy to possess a parasporal body within the exosporium (Figure 4). The functional significance of spore-associated crystals is not totally understood however benefits such as protection against UV degradation and better access to the target organism have been attributed to crystals attached within an exosporium [11]. If this is true, and the caps are shown to contain useful toxins, then those isolates showing the capped morphology may eventually prove useful in field applications. Clearly, more attention needs to be given to this phenotype and its mechanism of expression. For example, if the phenotype is plasmid encoded, could strains that undesirably liberate crystals be transformed to enclose their crystals within a protective cap?

Examples of filamentous appendages associated with bacterial spores are relatively rare where only a few isolates of *Clostridium *and *Bacillus *are reported to possess spores with appendages [12,13]. Even rarer is the association of an appendage with a parasporal body, as was observed with both Bt1-88 and Bt2-56. Very little is presently known about Bt1-88 and studies are underway to characterize both the strain and the ultra structure of the appendage-associated complex. From preliminary studies undertaken with Bt2-56, this isolate clearly demonstrates an intimate relationship between the filament and the parasporal body where the parasporal body appears to help anchor the filament to the spore (Figure 4 and [8]). Both these isolates raise a number of questions fundamental to our basic understanding of bacteria and the role of spore associated appendages. Among these is the obvious question of a possible relationship between the appendage and *B. thuringiensis *crystals, i.e., did some crystals evolve to act as an anchoring structure for spore associated filaments and if so, do they still retain any toxin activity? Similarly, what is the role of the filament and does it aid in pathogenicity?

Sequence data indicated that a gene in Bt1-33 is highly homologous to the *cry14Aa*1 gene, [GenBank: U13955] which has demonstrated activity against nematodes [14]. It was surprising that this relatively small study would produce an isolate containing a putative nematocidal gene when a much larger study utilizing the same primers did not [15]. However the gene in Bt1-33 is probably non-functional due to a frame-shift mutation, raising the question of why would it be maintained by the bacterium? One explanation could be that the gene is clustered in the genome with other genes necessary for pathogenicity as part of a pathogenicity island, and is being maintained in the genome indirectly through selection for the other genes in the group. Interestingly, PS80JJ1 (the strain gene U13955 is found in), has been shown to contain at least two other δ-endotoxin proteins Cry34Aa1 and Cry35Aa1, which together form a binary toxin shown to be active against *Diabrotica virgifera *(western corn rootworm). The corn root worm, like the nematode, is a soil-dwelling organism that *B. thuringiensis *are rarely active against. It is perhaps significant that U13955 is associated with other toxins that target a soil-dwelling organism, raising the question of whether PS80JJ1 is adapted against soil-dwelling organisms. If so, then perhaps like PS80JJ1, Bt1-33 may also possess other similarly rare toxin genes that target soil-dwelling organisms.

Using the PCR amplified fragment from the *cry14Aa*1-like gene in Bt1-33 as a probe, another isolate, Bt1-35, showed a weaker but definite hybridization signal. Perhaps significant is that it was isolated from the same sample as Bt1-33. The sequence of this gene is presently unknown however the reduced hybridization signal shown suggests that it may be an evolutionary relative or even share limited sequence, such as a functional domain.

The isolation method used in this study diverged from many of the reported methods for *B. thuringiensis *isolation by utilizing a stain instead of Phase Contrast Microscopy (which is commonly used when screening for *B. thuringiensis*), for the identification of crystals [1,16,17]. Although Phase Contrast Microscopy was useful for observing inside spore caps, the stain uniquely allowed a fast, high throughput evaluation of bacterial colonies for the presence of crystals. Similarly, the high contrast of the stain allowed it to identify relatively small crystals or low numbers of caps in a specimen. It is doubtful that without these benefits of the stain that Bt1-88, Bt1-33/35 and the relatively small crystal producers would have been isolated. This study also sought to reduce the chance of excluding some isolates by not incorporating overt selection during the isolation of candidate bacteria (e.g., not using antibiotics in the culture medium). The importance of reducing selective pressures for the successful isolation of each strain was not determined. However, at least one of the important isolates, Bt1-33, was isolated using Method G, a selective method. Thus it is possible that selective enrichment coupled with the stain and high throughput screening could have resulted in the isolation of higher numbers of *B. thuringiensis *candidates than were obtained in this study. Similarly, the isolation of capped isolates, including Bt2-56, could not be directly attributed to the use of the stain or even the general isolation methodology used. For example, although not as evident as with the use of the stain, Phase Contrast Microscopy could be used to visualize the capped spores. Thus it is possible that the high level of capped spores observed is best attributable to their high or unique presence in the Trinidadian environment. For example, it is difficult to imagine how the cap-associated filament of Bt2-56 has not previously been identified elsewhere, when in this screen it was isolated from several specimens collected throughout Trinidad. However, the filament was not easily observed under Phase Contrast Microscopy without some special attention being given to finding it. For example, the filament was not observed immediately with Phase Contrast Microscopy and it was only by chance that, as a capped spore, Bt2-56 was chosen for more intense study and its filament found. Similarly, the spiked structures associated with Bt1-88 were not observed on primary isolation or initial examination with Phase Contrast Microscopy, and it was only through a re-screening of all capped spores for filaments similar to Bt2-56 that it was observed. These data would suggest that some capped spores contained in existing *B. thuringiensis *libraries might also possess appendages and should be re-examined specifically for their presence.

Perhaps most striking about this screen was the low percentage of isolates producing bipyramidal crystals. In literally all reported screens for environmental isolates of *B. thuringiensis*, the percentage of bipyramidal crystals is usually above 40% [18,1], however only 3 isolates or 3.8 % of the non-clonal isolates where shown to have a bipyramidal morphology indicating a screen that deviated significantly from the norm.

## Conclusion

Results suggest that there are advantages to using a stain over the commonly used Phase Contrast Microscopy, particularly in the detection of small crystals, low numbers of crystals/capped spores and for its applicability for use in the fast, high throughput detection of *B. thuringiensis *candidates from the environment. There is a continuing need to explore the benefits of new methodologies for the detection of *B. thuringiensis *from the environment.

## Methods

### Sample collection and preparation

Samples were randomly collected from environmentally diverse sources (including beach sand, forest soil, aquatic and intertidal sediments, and soils from urban, rural and agricultural areas), placed in a plastic bag and held at room temperature until processing. Seven different methods (A-G) were initially used for sample preparation (Table 2), where Method F was used predominately (Table 3).

### *B. thuringiensis *isolation

Samples (0.25 g) were prepared using one or more of Methods A-G (Table 2), then 200 μl and 20 μl were cultured on Sporulation plates (Nutrient Agar with CCY salts [19] to which antibiotics were added as indicated in Table 2) at 30°C for 2 days to allow sporulation. Using a dissecting microscope and straight wire, an individual colony that conformed to the '*Bacillus cereus *group' (i.e. white to off-white with a matt appearance and irregular edges) was spotted onto a microscope slide that was pre-gridded with a wax pencil into 60 rectangles, and then stored by stabbing a grid on a nutrient agar plate. The agar plate was placed at 4°C to minimize germination of the spores. Where more than one isolate from the same sample was observed possessing the same crystal morphology, they were considered clonal and not all such isolates were kept. The slide was heat fixed and stained with Coomassie Brilliant Blue [7] and viewed under Brightfield Microscopy using a 100X immersion oil objective. Cell morphology was noted for all candidates of *B. thuringiensis*, which was defined as a large spore-forming rod whose ellipsoidal spore did not swell the mother cell and which produced a parasporal body upon sporulation [20,21].

### SDS protein electrophoresis

Two loopfuls of bacterial growth were removed from a Sporulation plate to 50 μl of ice cold Protein Sample Buffer [22], boiled for 2 min then frozen until use. Denaturing SDS gel electrophoresis was performed on protein samples according to the method of Lamelli [23]. The relative mobility for each band was calculated by applying the equation RH = A-C/A-B where A was the position of the top of the gel, B was the position of the top of the dye front and C was the position of the top of each band of interest. Relative mobility *vs *the molecular weights of known proteins (116 kDa, 92 kDa, 66 kDa, 45 kDa, 21 kDa) was plotted and linear regression performed to determine the equation for the line of best fit, which was used to determine the Molecular weights (KDa) of unknown bands.

### PCR of toxin genes

DNA template was prepared by lysis precipitation wherein approximately half a loopfull of cells from a sporulated plate was placed into 2 mls of L- Broth, incubated at 60°C for 10 min, then at 30°C with shaking at 250 rpm for approximately 5 h. Harvested cells were washed in water, resuspended in 200 μl of fresh lysing solution (Sodium duodecyl sulphate 3.3%; Tris 0.05 M, pH 12.5), incubated for 30 mins at 60°C and vortexed for 10 s to shear the DNA. DNA was collected by ethanol precipitation [24] and resuspended in 60 μl of 0.1 × TE buffer pH8.0. One microliter was used as template in PCR reactions. Cry and Cyt groups targeted, protocols and control strains utilized are given in Table 4. The ability of template preparations to be amplified in a PCR reaction was assessed using Bacillus 16S ribosomal RNA primers (Table 4), at 94°C for 15 sec; 55°C for 30 sec and 72°C for 2 min for 28 cycles with 1.5 mM MgCl.

### Hybridization of cry genes

Probe hybridization was performed using the DIG High Prime DNA Labeling and Detection Starter Kit (Roche Molecular Biochemicals, Cat# 1 745 832). *cry *and *cyt *gene probes were made by PCR (as described above) using the following bacterial strains from the Bacillus Genetic Stock Center (BGSC) as templates: *cry1*- BGSC4J3; *cry2*- BGSC4J3; *cry3*- BGSC4AA1; *cry4*- BGSC4Q1; *cry5*- Bt1-33; *cyt1*- BGSC4Q1; *cyt2*- BGSC4Q1; *16S*- BGSC4J3

### DNA sequencing

An ABI Cycle Sequencing Big Dye Ready Reaction kit (Catalogue # 4303500) was used according to the manufacturer's instructions and reactions resolved on an ABI 377 DNA Sequencing Machine. Primers gral-nem(d) and gral-nem(r) [15] were used to amplify and sequence the putative nematocidal gene in Bt1-33.

### Bioassay (quick screen)

Larvae of *Spodoptera frugiperda *were collected from a corn field and used to generate up to 5 generations of larvae. The width of the head capsule was used to select 5^th ^instar larvae for use in the bioassay [25]. Larvae were starved for approximately 1 hour immediately prior to being challenged with one loopful of a bacterial isolate collected from a sporulated colony and smeared onto a 1 cm^2 ^leaf piece (*Sorghum halipense*) in triplicate. Larvae were left for approximately 10 h then presented with a fresh piece of uninoculated leaf then left for a further 10–12 h. Scoring was: not eaten a score of 3; partially eaten a score of 2; completely eaten a score of 1. The toxicity value of a *B. thuringiensis *candidate was the average of the scores for the three trials, which were arbitrarily classified as: 1.0- not toxic; > 1.0 but < 2.0 – uncertain toxicity; = 2.0- toxic. All isolates in the library were tested, irrespective of whether they were considered clonal strains, as well as the five control strains obtained from the Bacillus Genetic Stock Center.

## Authors' contributions

JR was principally responsible for the design and execution of the project and data analysis. DA contributed to project design and data analysis. Both authors read and approved the final manuscript.

## Figures and Tables

**Table 1:** Isolates positive for *cry *and *cyt *genes as determined by Probe Hybridization and PCR.

**Sample number**	**Isolate #**	***cry1***	***cry1 *C**	***cry1 *D**	***cry2***	***cry 3,7,8***	***cry4***	***cry5*/Nem**	***cyt1***	***cyt2***
22	Bt1-33							H-P		
22	Bt1-35							H		
72	Bt2-46	H-P			H-P					
81	Bt2-48				P					
N/A	*Bt2-50*	**H-P**	P	P	H-P					
N/A	*Bt2-51*	**H**			**H-P**					H
N/A	*Bt2-52*	**H-P**	**P**	P	**H-P**					
N/A	*Bt2-53*						**H-P**			***
N/A	*Bt2-54*						**H-P**		**H-P**	H
N/A	*Bt2-55*					**H-P**				***
40	Bt1-65	P					H-P			
58	Bt1-74						H			
63	Bt1-83	H								
11	Bt1-1						P			
11	Bt1-4						P			
11	Bt1-5						P			
15	Bt1-6						P			
41	Bt2-14						P			P
41	Bt2-15						H-P			
71	Bt2-21									H
77	Bt2-24								P	
82	Bt2-35								P	

(H) denotes probe hybridization and (P) PCR. Samples Bt2-(50–55) were control strains where bold type denotes genes that strains are reported to contain, and (***) designates genes that are reported to be present but were not detected.

**Table 2:** Summary of preparation methods A-G

Method	Key points
A	Sample heated for 15 min in Nutrient Broth at 65°C, then plated on Sporulation plates with 20 IU/ml penicillin G
B	4 hour Pre-incubation of sample in L-broth (30°C shaking at 250 rpm) then heated for 15 min at 65°C then plated on Sporulation plates
C	Same as Method B but plated on Sporulation plates with 20 IU/ml penicillin G
D	4 hour Pre-incubation of sample in L-broth/acetate [26] (30°C shaking at 250 rpm) then heated for 15 min at 65°C then plated on Sporulation plates
E	Same as Method D above except plated on Sporulation plates with 20 IU/ml penicillin G
F	Sample heated for 15 min in L-broth at 65°C then plated on Sporulation plates
G	Same as Method F above except plated on Sporulation plates with 20 IU/ml penicillin G

**Table 3:** Preparation methods and their use.

**Preparation Method**	**Number of samples Screened**	**Number of Colonies Screened**	**Number of Samples with *B. thuringiensis *Isolates**
A	13	1,560	1
B	2	120	0
C	2	54	1
D	2	80	0
E	2	34	1
F	70	7,172	43
G	13	1,329	4
	**Total: 104**	**Total: **10,349	**Total: **50

**Table 4:** Primers and probes utilized.

**Target Gene**	**Reference**	**Control Strains**
***cry1 Family***	Juarez-Perez et al., [27]	BGSC 4J3
***cry1C***	Juarez-Perez et al., [27]	BGSC4J3
***cry1D***	Juarez-Perez et al., [27]	BGSC 4J3
***cry2 Family***	Masson et al., [28]	BGSC 4J3
***cry3, 7, 8 *** **Family**	Ceron et al., [29]	BGSC4AA1
***cry4***	Ben Dov et al., [6]	BGSC4Q1
***cry5, 12, 14, 21 *** **Family**	Bravo et al., [15]	none available
***cyt1***	Bravo et al., [15]	BGSC 4Q1
**Bacillus 16S *rDNA***	Siefert et al., [30]	Not Applicable

(H) denotes probe hybridization and (P) PCR. Samples Bt2-(50–55) were control strains where bold type denotes genes that strains are reported to contain, and (***) designates genes that are reported to be present but were not detected.
